# Arabidopsis AN3 and OLIGOCELLULA genes link telomere maintenance mechanisms with cell division and expansion control

**DOI:** 10.21203/rs.3.rs-3438810/v1

**Published:** 2023-10-28

**Authors:** Inna A. Agabekian, Liliia R. Abdulkina, Alina Y. Lushnenko, Pierce G. Young, Lia R. Valeeva, Olivia Boskovic, Ethan G. Lilly, Margarita R. Sharipova, Dorothy E. Shippen, Thomas E. Juenger, Eugene V Shakirov

**Affiliations:** Kazan Federal University: Kazanskij Privolzskij federal’nyj universitet; Kazan Federal University: Kazanskij Privolzskij federal’nyj universitet; Kazan Federal University: Kazanskij Privolzskij federal’nyj universitet; Texas A&M University; Marshall University; Marshall University; Marshall University; Kazan Federal University: Kazanskij Privolzskij federal’nyj universitet; Texas A&M University; The University of Texas at Austin; Marshall University

**Keywords:** Arabidopsis thaliana, NOP2, ribosomal proteins, telomere dynamics, telomerase, tert

## Abstract

Telomeres are conserved chromosomal structures necessary for continued cell division and proliferation. In addition to the classical telomerase pathway, multiple other genes including those involved in ribosome metabolism and chromatin modification contribute to telomere length maintenance. We previously reported that *Arabidopsis thaliana* ribosome biogenesis genes *OLI2/NOP2A, OLI5/RPL5A* and *OLI7/RPL5B* have critical roles in telomere length regulation. These three *OLIGOCELLULA* genes were also shown to function in cell proliferation and expansion control and to genetically interact with the transcriptional co-activator *ANGUSTIFOLIA3 (AN3)*. Here we show that *AN3*-deficient plants progressively lose telomeric DNA in early homozygous mutant generations, but ultimately establish a new shorter telomere length setpoint by the fifth mutant generation with a telomere length similar to *oli2/nop2a* - deficient plants. Analysis of double *an3 oli2* mutants indicates that the two genes are epistatic for telomere length control. Telomere shortening in *an3* and *oli* mutants is not caused by telomerase inhibition; wild type levels of telomerase activity are detected in all analyzed mutants *in vitro*. Late generations of *an3* and *oli* mutants are prone to stem cell damage in the root apical meristem, implying that genes regulating telomere length may have conserved functional roles in stem cell maintenance mechanisms. Multiple instances of anaphase fusions in late generations of *oli5* and *oli7* mutants were observed, highlighting an unexpected effect of ribosome biogenesis factors on chromosome integrity. Overall, our data implicate AN3 transcription coactivator and OLIGOCELLULA proteins in the establishment of telomere length set point in plants and further suggest that multiple regulators with pleiotropic functions can connect telomere biology with cell proliferation and cell expansion pathways.

## Introduction

For eukaryotic cells to maintain genomic stability, the physical ends of their linear chromosomes require protection from being recognized as double-strand breaks and subsequent illegitimate end-to-end fusions. Telomeres consist of highly repetitive DNA sequences bound by dedicated proteins that provide such protection by capping chromosomes and promoting successful DNA replication through the DNA terminus to facilitate cell proliferation (Bonnell et al. 2021). The length of telomeric DNA tract is established and maintained at a species-specific set point, though it varies widely among different organisms and even among populations of the same species (Shakirov et al. 2022). While many genes are known to control telomere length homeostasis, our current understanding of the genetic and molecular mechanisms that establish the initial telomere length set point remains limited. Genome-wide studies in multiple species implicated hundreds of genes in telomere length regulation (Askree et al. 2004; Codd et al. 2013; Li et al. 2020; Choi et al. 2021). Recent evidence also indicated that some of these genes function in telomere length control indirectly and suggested that functional crosstalk occurs between mechanisms that modulate telomere length homeostasis, chromatin modification, rRNA biogenesis, cell proliferation and other physiological and developmental pathways (Xie and Shippen, 2018; Wang et al. 2023; Shakirov et al. 2022).

Using QTL mapping and transgenic manipulations in the model plant *Arabidopsis thaliana*, we recently identified *OLI2/NOP2A*, which encodes a ribosomal RNA methyltransferase, as a novel positive regulator of telomere length (Abdulkina et al. 2019). Plants deficient in the *OLI2/NOP2A* lose up to 27% of their telomeric DNA. By the third mutant generation (G3) these plants establish a new shorter telomere length set point that is stable for at least three subsequent generations. The human homolog of Arabidopsis *OLI2/NOP2A*, dubbed *NOP2* (Bourgeois et al., 2015), functions as a S-adenosyl-L-methionine dependent methyltransferase in ribosome biogenesis, cell proliferation, and cancer progression (Freeman et al. 1988; Ranzani et al. 2013). Notably, the A*. thaliana OLI2/NOP2A* was previously identified in a forward genetic screen for genes involved in coordination of cell proliferation and cell expansion (Fujikura et al. 2009). Besides *OLI2/NOP2A*, several other *OLIGOCELLULA (OLI)* genes were identified, including *OLI5/RPL5A* and *OLI7/RPL5B*, which encode the Arabidopsis paralogs of ribosomal protein L5 (Pinon et al. 2008). RPL5 binds specifically to 5S rRNA and serves as an essential structural component of the large 60 S ribosomal subunit (Mathieu et al. 2003).

Coordination of cell division and cell proliferation is a critical developmental process. In Arabidopsis, mutations in a group of genes, including *ANGUSTIFOLIA3 (AN3)*, result in a decreased number of leaf cells and lead to a significant postmitotic increase in the size of remaining cells to maintain nearly normal leaf area, a phenomenon known as “compensation” (Nozaki et al. 2020). For example, in *an3* mutant leaves, the number of subepidermal cells drops to ~ 30% of wild type, but these plants compensate for lost leaf area by increasing the size of the remaining cells to 150% of those in the wild type (Horiguchi et al. 2005). Thus, cell proliferation and cell expansion are highly coordinated events necessary to allow proper organ development (Horiguchi et al. 2011). *AN3*, also known as *Arabidopsis GRF-INTERACTING FACTOR1 (AtGIF1),* is a transcriptional co-activator that promotes cell proliferation. Its homologs are also involved in ribosomal biogenesis in dicots (Vercruyssen et al. 2014) and modulate transformation efficiencies in monocots, possibly through roles in stem cell maintenance (Lee and Wang, 2023). *AN3* encodes a homolog of the human transcription co-activator known as synovial sarcoma translocation protein (SYT) (Brett et al. 1997; Horiguchi et al. 2005). Both SYT and AN3 coactivators interact with the chromatin remodeling SWI/SNF complexes (i.e., BRAHMA) to stimulate expression of a suite of cell proliferation and cell cycle genes (Vercruyssen et al. 2014; Yu et al. 2019).

Not all mutations that affect cell proliferation in leaves trigger compensation. Plants bearing single mutations in any one of the three *OLIGOCELLULA* genes (*OLI2, OLI5* or *OLI7*) do not exhibit compensation, despite showing up to 50% reduction in leaf cell numbers. However, combining any of these mutations with an3 leads to markedly enhanced compensation, implying that compensation is regulated by a threshold-dependent mechanism (Fujikura et al. 2009). Previous findings implicating *OLI2, OLI5* and *OLI7* in ribosome biogenesis further indicate that compensation and organ size more generally may be under dual regulation by *AN3* and ribosome-related processes.

We previously reported that several *OLI* genes affecting organ size control are involved in telomere biology and serve as positive regulators of telomere length (Abdulkina et al. 2019). Since *OLI2, OLI5* and *OLI7* control cell proliferation through their interaction with the *AN3*-dependent pathway, here we investigate the role of the *AN3* gene in telomere biology. Using a multigenerational analysis, we report that *A. thaliana an3* mutants display telomeres shorter than wild type. Moreover, double *an3 oli2* mutants harbor telomeres that are similar in size to the shortened telomeres in either single mutant, suggesting that *AN3* and *OLI2* genes act in the same genetic pathway for telomere maintenance. Although telomerase activity levels are wild type in all analyzed *an3* and *oli* mutant plants, late generations of these mutants display evidence of cell death in the root apical meristem, suggesting that a common mechanism may connect telomere length control to cell proliferation and viability. Overall, our data reveal a novel set of genetic interactions governing control of telomere length and cell division in plants.

## Results

### Inactivation of the AN3 gene leads to shorter telomeres in A. thaliana.

**Figure 1:**
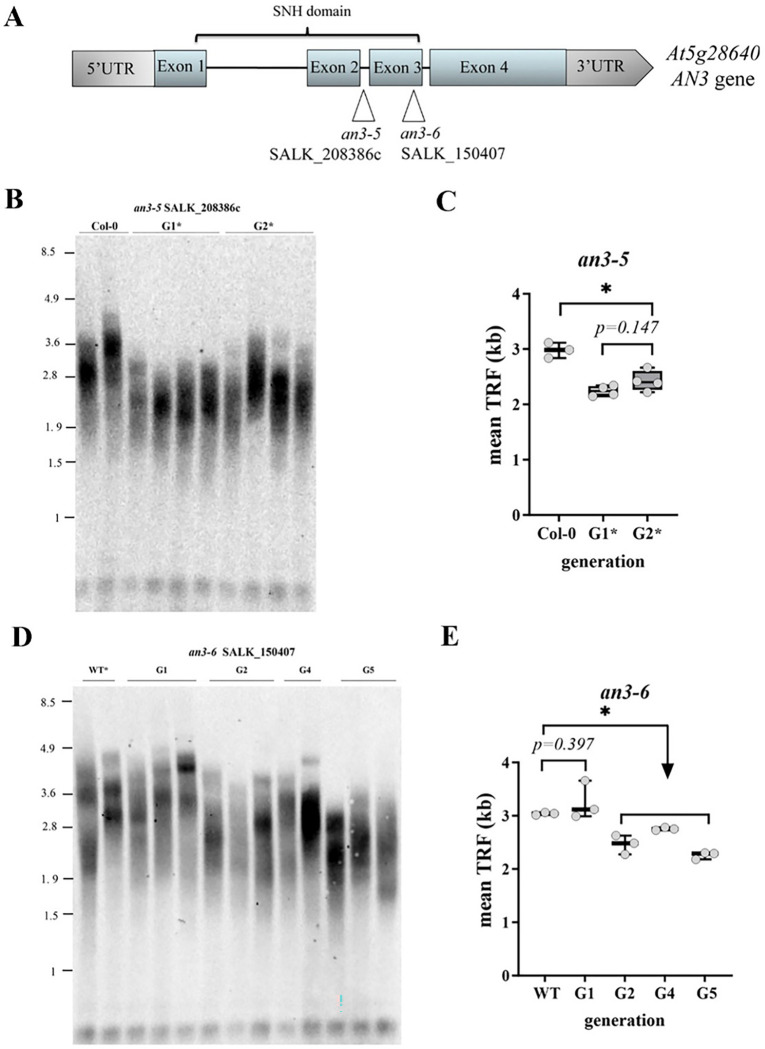
Inactivation of Arabidopsis *AN3* gene leads to the establishment of a new shorter telomere length set point. **(A)** A diagram showing the *Arabidopsis AN3* gene model. Blue boxes represent exons, grey boxes represent untranslated regions, lines represent introns. Relative positions of T-DNA insertion sites and the corresponding mutant line numbers are indicated. The conserved and functionally important synovial sarcoma associated SYT N-terminal homology (SNH) domain is indicated on top. (**B**) TRF southern blot for Col-0 wild type and plants from two consecutive generations of *an3–5* homozygous T-DNA mutants. Asterisks denote mutant plant generations that were propagated in the lab. Molecular weight DNA markers (in kb) are shown on the left. (**C**) Telomere length (mean TRF) distributions in ≥3 biological replicates of each plant genotype and generation are shown in boxplots. Data points represent mean TRF values from individual plants (biological repeats) analyzed with TeloTool. Error bars indicate minimum to maximum values. Significance codes based on unpaired t-test with Welch correlation: *P ≤ 0.05. (**D**) A representative TRF blot for telomere length analysis of several consecutive generations of homozygous mutant plants segregated from self-pollinated heterozygous *an3–6*^*+/−*^ parent. (E) Telomere length (mean TRF) distributions in ≥3 biological replicates of each *an3–6* mutant generation are shown in boxplots.

To evaluate the potential role of *AN3* in telomere biology, we measured mean telomere length by the terminal restriction fragment (TRF) assay in two different lines of an3 mutants (Fig. 1A), and quantitated the results using the TeloTool program (Gohring et al. 2014). We first analyzed telomere length in SALK_208386c mutants (designated hereafter *an3–5*). These seeds were obtained from the seed stock center in the confirmed homozygous mutant state and thus were likely propagated for several generations in the absence of AN3 protein prior to our analysis. Two consecutive generations of *an3–5* mutants showed shorter mean telomere length compared to the Col-0 wild type control (Fig. 1B), with an average shortening of approximately 650 bp over wild type levels (Fig. 1C). Notably, we did not detect a significant telomere size difference between these two mutant generations (Fig. 1C), indicating that telomere length in *an3–5* mutants was established at a shorter, but stable, equilibrium.

To test how many plant generations are required to reach this length set point, we analyzed a second *an3* mutant T-DNA line (SALK_150407), designated hereafter *an3–6*. TRF analysis was performed on sibling plants segregated from self-pollination of a heterozygous *an3–6*^*+/−*^ parent. Interestingly, telomere length in segregants of the first true *an3–6* mutant generation (G1) was 3,257 bp and not significantly different from wild type siblings (Fig. 1D, E), implying that telomere loss is not immediate and may occur in later mutant generations. Indeed, mean telomere length in the second homozygous *an3–6* mutant generation (G2) decreased to ~ 2.5 kb (Fig. 1D, E). Telomere length slightly fluctuated in the next mutant generations, but were generally maintained in the 2.2–2.7 kb size range through at least the fifth mutant generation (G5). This newly established mean telomere length range is similar for *an3–6* and *an3–5* mutants, suggesting that later generations of *an3* mutants maintain telomeres at a set point that is 16.6% shorter than in the wild type. We conclude that the *AN3* gene is a positive regulator of Arabidopsis telomere length.

### AN3 and OLI2/NOP2A mutants have similar effects on telomere length.

Both *AN3* and *OLI2/NOP2A* were previously implicated in cell proliferation (Fujikura et al. 2009), and our data suggest that both genes are also involved in telomere length control (this study; Abdulkina et al. 2019). We thus asked if *AN3* and *OLI2/NOP2A* function in the same or different genetic pathways for telomere length regulation. Specifically, we hypothesized that if *AN3* functions in a pathway separate from *OLI2/NOP2A*, the addition of the *an3–6* mutation would increase the rate of telomere shortening observed in *oli2–4* mutant background. To test this prediction, we generated double heterozygous F1 progeny, and propagated them to F2 to obtain individual mutant genotypes and wild type controls (Supplemental Fig. 1).

**Figure 2:**
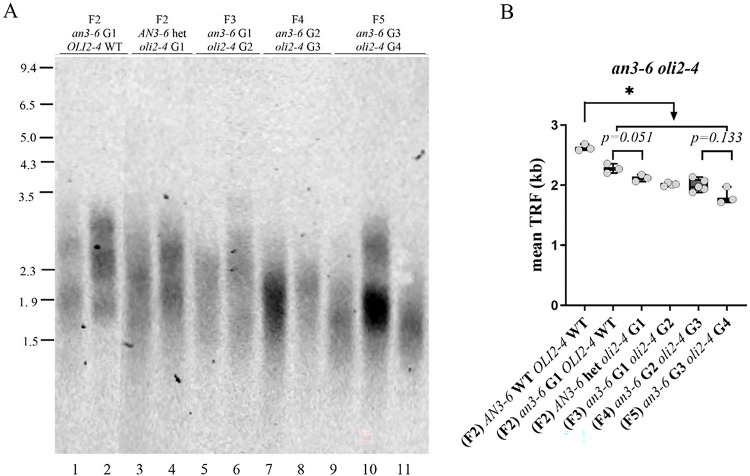
*AN3* and *OLI2* likely act in the same genetic pathway for telomere length regulation. **(A)** A representative TRF blot for F2-F5 generations of double mutant *an3–6 oli2–4* plants. Molecular weight DNA markers (in kb) are shown on the left. (**B**) Telomere length (mean TRF) distribution in ≥3 biological replicates of each analyzed mutant genotype and generation are shown in boxplots. Data points represent mean TRF values from individual plants (biological repeats) analyzed with TeloTool. Significance codes based on unpaired t-test with Welch correlation: *P ≤ 0.05.

We first compared telomere length in F2 siblings that were homozygous mutants for either *AN3* or *OLI2*. Telomere length in G1 *an3–6*^*−/−*^ plants was not significantly different from that observed in G1 *oli2–4*^*−/−*^ mutants (which in our setup were also heterozygous for *an3–6*^*+/−*^ mutation) (Fig. 2A, compare lanes 1,2 and 3,4; Fig. 2B). Since we could not obtain double homozygous *an3–6*^*−/−*^ *oli2–4* ^*−/−*^ mutants in the F2 generation, we self-pollinated *an3–6*^*+/−*^ *oli2–4*^*−/−*^ plants to generate F3 mutants that represent the first generation of double homozygous mutants *oli2–4* ^*−/−*^ (G2) *an3–6*^*−/−*^ (G1). Notably, these F3 double mutants displayed telomeres that were only slightly shorter relative to their *oli2–4*^*−/−*^ (G1) *an3–6*^*+/−*^ F2 parents (Fig. 2B; 2,010 bp vs 2,117 bp, respectively). This finding suggests that the *an3–6* mutation does not make a significant contribution to the rate of telomere shortening observed in *oli2–4*^*−/−*^ mutants. Consistent with this observation, telomere length in the following two generations (F4 and F5) of the double mutants *an3–6*^*−/−*^ *oli2–4*^*−/−*^ continued to fluctuate in the ~ 1.9–2 kb range (Fig. 2A,B), which is characteristic of the late generations of *oli2–4*^*−/−*^ mutants (Abdulkina et al. 2019). These data imply that the rate of telomere length shortening in the double *oli2–4* ^*−/−*^ *an3–6*^*−/−*^ mutants is similar to the single *oli2–4* mutant. We surmise that *AN3* and *OLI2/NOP2A* genes, despite both being positive regulators, do not show synergistic effects on telomere length.

### Mutations in AN3 or the OLIGOCELLULA cell proliferation genes do not affect telomerase activity.

**Figure 3:**
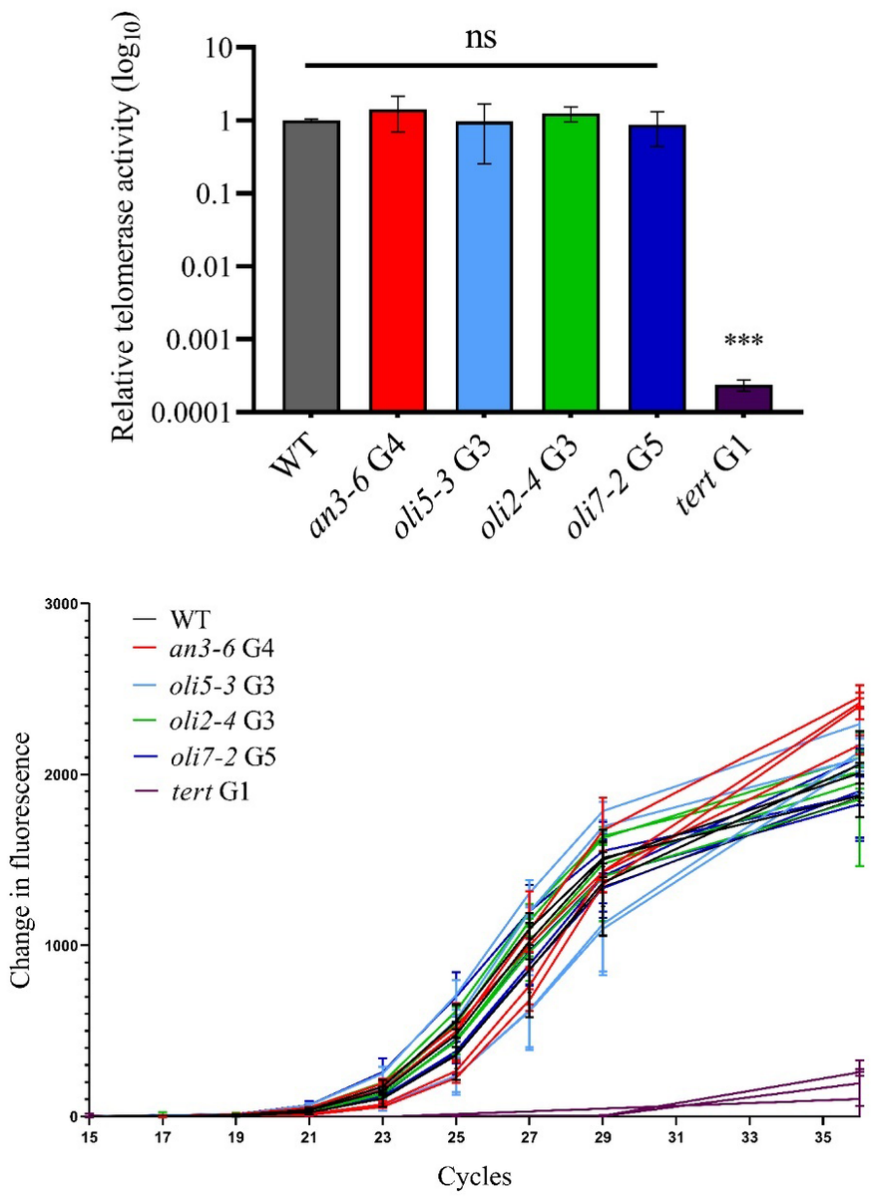
Relative telomerase activity in *an3* and *oli* mutants is similar to the wild type level *in vitro.* The top panel represents a histogram of the relative telomerase activity detected by real-time TRAP in flower bundles of *an3–6, oligocellula* mutants *oli5–3, oli2–4, oli7–2,* wild type Col-0 and the telomerase *tert* mutants (negative control). The mean of ≥3 biological replicates for each plant genotype is shown as a log_10_ of fold change compared to wild type. Error bars indicate standard deviation. Significance codes based on Students t-test: NS: P > 0.05, ***P ≤ 0.001. The bottom panel shows fluorescence change data for four individual biological replicates of the wild type, *an3–6* and *oli* mutants and three biological replicates of the *tert* mutants.

Given that mutations in the *AN3* and *OLIGOCELLULA* genes (*OLI2/NOP2A, OLI5/RPL5A* and *OLI7/RPL5B*) result in a shorter telomere phenotype, we considered the possibility that these plants have reduced levels of telomerase activity. To measure telomerase activity, we performed quantitative real-time telomeric repeat amplification protocol (qTRAP) on flowers (Song et al. 2019). This assay measures enzyme activity levels using protein extracts that are rich in telomerase (Fitzgerald et al. 1999). As for wild type plants, we detected robust telomerase activity in all samples prepared from *an3–6* mutants (Fig. 3). Likewise, no significant changes in telomerase activity were detected in extracts from *oli5–3, oli2–4*, and *oli7–2* mutants (Fig. 3), indicating that inactivation of these genes does not affect telomerase activity levels in vitro. We conclude that telomere shortening in *an3, oli2/nop2a, oli5/rpl5a* and *oli7/rpl5b* mutants is not caused by biochemical changes in the telomerase enzyme activity.

### Late generation AN3 and OLIGOCELLULA mutants display evidence of cell death in the root apical meristem.

**Figure 4:**
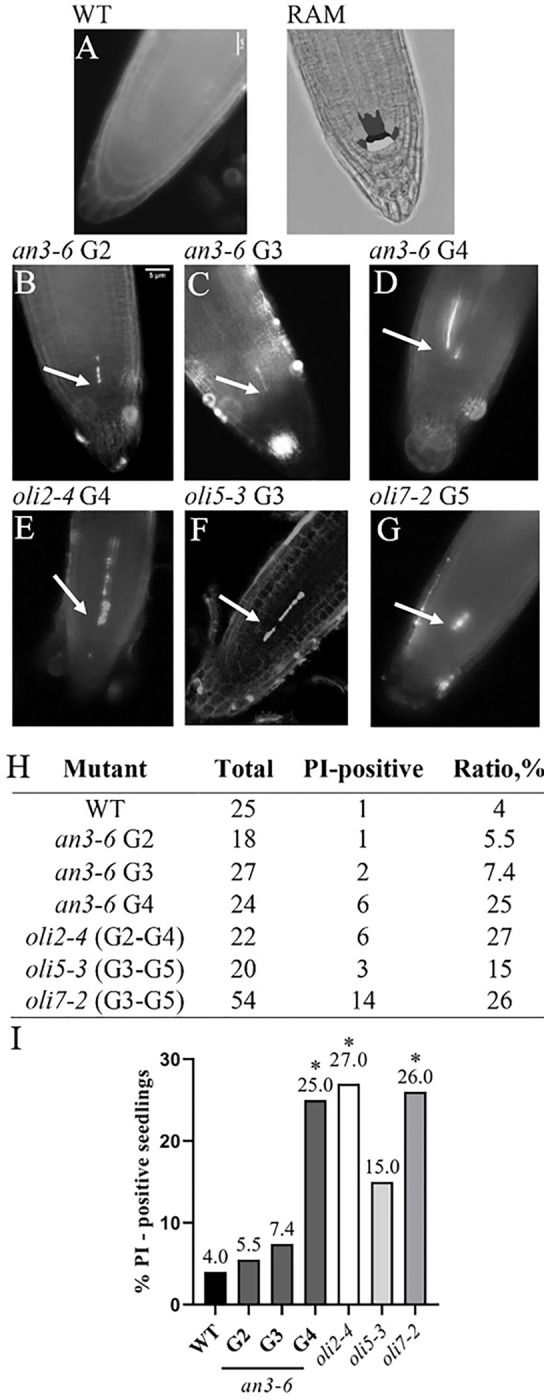
Analysis of root tips of wild type, *an3* and *oli* mutant plants stained with propidium iodide. **(A-G)** Representative images of root tips of 5-day-old WT (**A**), *an3–6* G2 (**B**), *an3–6* G3 (**C**), *an3–6* G4 (**D**), *oli2–4* G4 (**E**), *oli5–3* G3 (**F**) and *oli7–2* G5 (**G**) mutant seedlings stained with PI. Arrows point to PI-positive cells in the quiescent center of root meristems in *an3–6* and *oli* mutants. For reference, a scheme of the root tip is shown on the top (RAM). Gray cells in the root apical meristem (RAM) indicate the stem and precursor cells around the quiescent center (QC) (in white). Numbers of PI-positive roots (**H**) and graphical representation (**I**) of the percentages of PI-positive roots for wild type and mutants are shown. *P ≤ 0.05 based on Fisher exact test.

Mutations in major regulators of chromosome maintenance and cellular proliferation, as well as telomere length defects, can lead to genome damage and cell death in the root apical meristem (Xie and Shippen, 2018; Surovtseva et al. 2009, Wang et al. 2023). Therefore, we looked for evidence of cell death in three consecutive generations of *an3–6* mutants by performing propidium iodide (PI) staining of root cells. PI stains nucleic acids of dead cells but is eliminated from live cells (Amiard et al. 2010). For wild type root apical meristems (RAM), 4% of the roots we monitored contained PI-positive cells (Fig. 4A). Similarly for G2 and G3 *an3–6* plants 5.5% and 7.4% of the roots we examined contained PI-positive cells (Fig. 4B,C). However, the number of PI-positive cells jumped dramatically to 25% for G4 *an3–6* mutants (Fig. 4D,H). This surge in PI staining in *an3–6* plants correlates with the establishment of the new shorter telomere length set point in late generations of this mutant. We next tested if the increase in PI staining is specific to *AN3* mutants or can also be observed in plants with mutations in *OLIGOCELLULA* genes. We found that roots of all analyzed late-generation *OLIGOCELLULA* mutants (*nop2a/oli2–4, rpl5a/oli5–3*, and *rpl5b/oli7–2*) harbor an increased number of PI-positive RAMs compared to wild type (Fig. 4E–I). The correlation between shorter telomeres and increased meristematic cell death in late generations of *an3–6, nop2a/oli2–4, rpl5a/oli5–3,* and *rpl5b/oli7–2* mutants suggests a common mechanism through which telomere length control is connected to cell proliferation and genome maintenance check points.

### OLI5 and OLI7 genes, but not AN3 or OLI2, contribute to chromosome stability.

**Figure 5:**
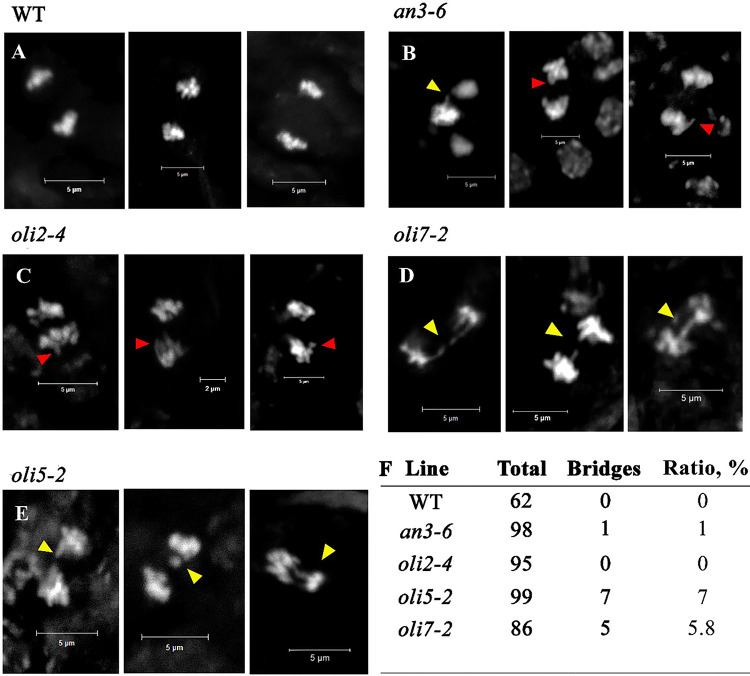
*OLI5* and *OLI7* genes contribute to chromosome maintenance. **(A-E)** Representative images of mitotic spreads from WT (**A**), *an3–6* G3 (**B**), *oli2–4* G3 (**C**), *oli7–2* G4 (**D**), *oli5–2* G3 (**E**) pistils. Cells were stained with DAPI and observed using a fluorescent microscope with 63X magnification. Red arrows indicate chromosome lagging, yellow arrows indicate anaphase bridges. (**F**) Frequencies of anaphase bridges observed for mitotic cells from each genotype are indicated.

Mutations in chromosome capping proteins or critical shortening of telomeric DNA tracts can lead to an increased incidence of end-to-end chromosome fusions and genome aberrations (Borges et al. 2022; Song et al. 2008). To check if telomere integrity is compromised in *an3* and *oli* mutants, we performed cytogenetic analysis of mitotically dividing cells in pistils. As expected, we saw no chromosomal segregation defects in wild type plants (Fig. 5A). Although we did detect rare incidences of lagging chromosomes (Guo et al. 2020) during anaphase in *an3–6* mutants, we found no evidence of anaphase bridges indicative of chromosome fusion in either *an3–6* or in *oli2–4* mutants (Figs. 5B and 5C). Notably, however, bridged chromosomes were associated with 5.8% and 7% of the anaphases in *oli7–2* and *oli5–2* mutants, respectively (Fig. 5D, E and F), consistent with a role for these genes in maintaining chromosome stability.

**Figure 6:**
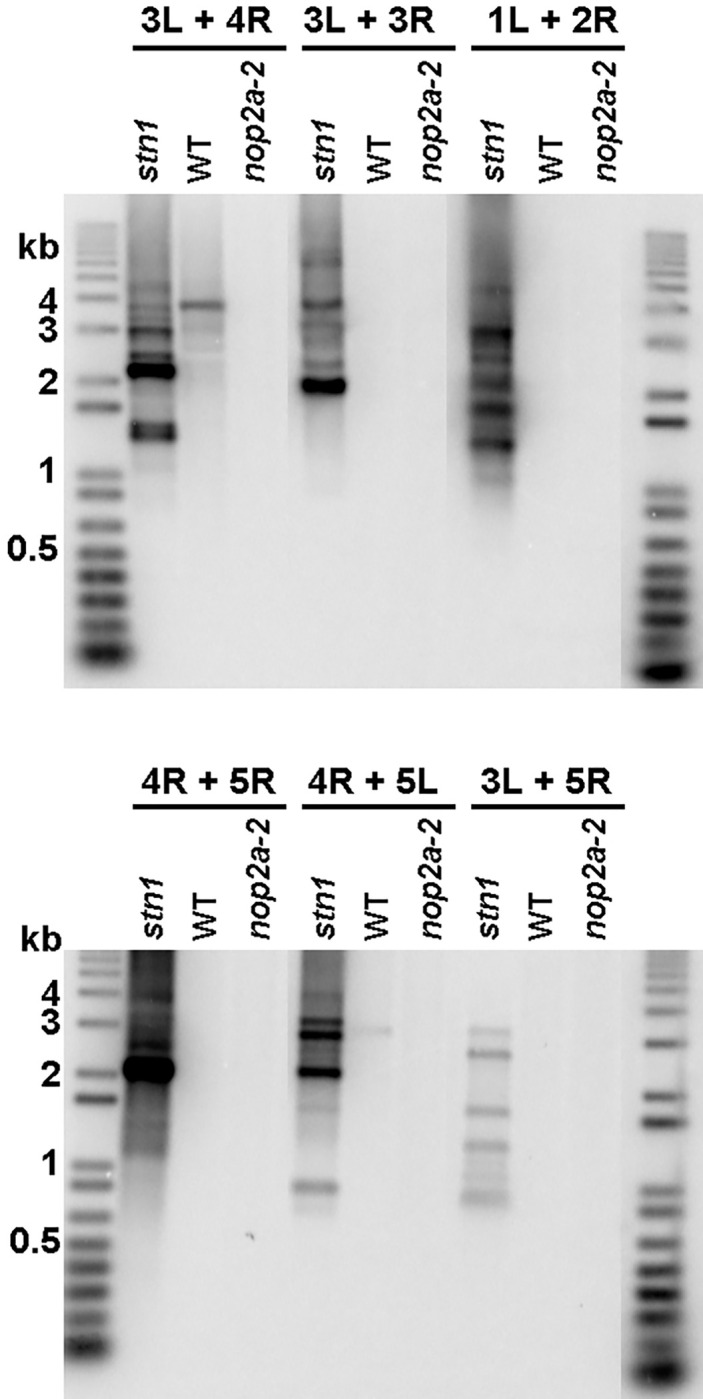
PCR amplification of telomeric end-to-end chromosome fusions using chromosome-specific subtelomeric primers. Amplification only occurs when two telomeric regions from different chromosome arms are covalently joined end-to-end. Southern analysis of fusion PCR products is then performed using a telomeric probe. Panels show Fusion PCR results for different subtelomeric primer combinations, using wild type (WT) template DNA (negative control), DNA from *stn1–1* (positive control) and *nop2a-2/oli2*-*2*-mutants. Results for a single plant are shown in each lane. The subtelomeric primers used for fusion PCR are indicated, i.e., 3L (left arm of chromosome 3) + 4R (right arm of chromosome 4). Molecular weight DNA markers (in kb) are shown.

To evaluate if the chromosome segregation defects observed in *OLIGOCELLULA* mutants correspond to telomere-to-telomere fusions, we performed telomere fusion PCR (TF-PCR), an assay that detects covalently joined telomeres using combinations of primers directed at unique subtelomeric sequences from different chromosome ends (Heacock et al. 2004). As positive and negative controls, we utilized stn1 mutants and Col-0 plants, respectively (Song et al. 2008). Abundant TF-PCR products were observed in stn1 mutants, but no or few TF-PCR products were detected with *oli2–2/nop2a-2* (Fig. 6) or with *an3–6, oli7–2* and *oli5–2* DNA (Supplemental Fig. 2). These results suggest that the chromatin bridges observed in *oli5* and *oli7* mutants may not result from telomere fusions and instead may represent aberrations involving other parts of chromosomes. This conclusion is consistent with previous observations for these mutants (Abdulkina et al. 2019) indicating that telomere length in *oli5* and *oli7* plants exceeds the critical minimal size threshold of 1.0 kb required to avert telomere-to-telomere fusions (Heacock et al., 2004). Taken together, our data indicate that *AN3* and *OLIGOCELLULA* genes are important for the establishment of telomere length set point and for some aspects of general chromosome stability in Arabidopsis, but are largely dispensable for chromosome end protection.

## Discussion

Much attention has recently been given to the non-canonical functions of telomerase and telomere-associated factors in DNA repair, transcription and cell cycle control (Barbero Barcenilla B, Shippen, 2019; Hong et al. 2016; Valeeva et al. 2023). Conversely, multiple lines of evidence from different eukaryotic systems indicate that many developmental and cellular pathways beyond the classical telomere biology genes also have pleiotropic impacts on telomere length regulation. Here we show that the transcriptional co-activator *ANGUSTIFOLIA3/ GRF-INTERACTING FACTOR1 (AN3/GIF1)*, besides its major role in chromatin remodeling and cell division control, functions as a positive regulator of Arabidopsis telomere length. Telomeres in two *an3* mutant lines shorten to a similar level as in plants deficient for the ribosome biogenesis *OLIGOCELLULA* genes, and ultimately establish a new shorter telomere length setpoint. Given the previously described genetic interactions between components of the *OLIGOCELLULA*-dependent rRNA maturation/ribosome assembly pathway and the *AN3*-dependent cell proliferation mechanisms (Fujikura et al. 2009), our data imply that telomere length homeostasis is controlled by an even larger scope of pleiotropic genetic networks than previously envisioned.

Considering the well-established roles of OLI2/NOP2A, OLI5/RPL5A and OLI7/RPL5B proteins in rRNA binding and biogenesis (Bourgeois et al. 2015; Fujikura et al., 2009), we asked if telomere shortening observed in these mutants could be due to decreased activity of the telomerase reverse transcriptase. However, the levels of telomerase activity *in vitro* remained unaltered in *an3* and all tested *oli* mutant plants, suggesting that the *OLIGOCELLULA* or *AN3* pathways are not involved in the maturation of the telomerase RNA subunit or in its stability *in vivo*. This observation is intriguing and suggests that other mechanisms could be responsible for telomere shortening observed in these mutants. Several possibilities can be envisioned. First, the telomerase holoenzyme can be regulated *in vivo* through its recruitment to the DNA substrate or through changes in its subcellular localization, including cell cycle-related trafficking (Robinson and Schiemann, 2022). Given the previously proposed role of AN3/GIF1 and other GRF-interacting factors in cell cycle regulation (Lee et al. 2009), the role of these proteins in regulating telomerase access to the telomere at different phases of the cell cycle is plausible. Other potential explanations for telomere shortening include deficiencies in telomeric DNA binding proteins that regulate telomerase access. Unfortunately, we still do not have a good understanding of the full complement of such factors in plants (Fulcher et al. 2015; Procházková Schrumpfová et al., 2016). Alternatively, defects in general protein synthesis that are characteristic of these ribosome biogenesis mutants may also impact telomere length indirectly (Garus and Autexier, 2021).

The degree of telomere length decline observed in *an3* and *oli* mutants is remarkably similar to that of Arabidopsis Replication Protein A (RPA) mutants (Aklilu et al., 2020). The *A. thaliana* trimeric RPA protein complexes have multiple roles in DNA replication and metabolism, including functions in telomere length control, but not in regulation of telomerase activity or in chromosome end protection (Audry et al., 2015). Interestingly, telomere length in several Arabidopsis *rpa* single and double mutants also initially declines but eventually stabilizes at a new shorter length equilibrium which is stably maintained through multiple subsequent mutant generations (Aklilu et al., 2020). The similarities in telomere phenotypes between *rpa*, *an3* and *oli* mutants raise an intriguing possibility that cell cycle and cell division control mechanisms can ultimately operate in close coordination with DNA replication and recombination machinery to sustain proper telomeric DNA length.

While the precise mechanism for telomere shortening is unknown, our analysis of the *an3–6 oli2–4* double mutant argues that *AN3* and *OLIGOCELLULA* genes genetically interact and likely participate in the same pathway for telomere length control. The data are also consistent with previous findings from genome-wide tandem chromatin affinity purification assays placing *OLI2* gene expression under *AN3* control (Vercruyssen et al. 2014). *AN3* and *OLIGOCELLULA* genes are known to functionally interact in cell size control, and combining mutations in any *OLIGOCELLULA* gene with *AN3* mutation leads to synergistic drop in leaf cell number and to markedly enhanced compensation in cell size (Fujikura et al. 2009). Curiously, double *an3–6 oli2–4* mutants do not display shorter telomeres than in either single mutant, suggesting that synergistic effects are specific to the function of these genes in cell proliferation, but not in telomere length control. These data further imply that the *AN3* and *OLIGOCELLULA* genes interact, but their genetic pathways are at least partially separate.

We also discovered that cells in the root apical meristems of late generation *an3* and all tested *oli* mutants are more susceptible to stem cell damage than wild type cells. This finding is consistent with other recent observations on the influence of telomere biology genes on stem cell biology. Stem cell renewal in the *Arabidopsis* RAM niche is regulated by several groups of genes, including SCARECROW and PLETHORA family members (Xiong et al. 2020). Expression of some of these genes is misregulated in *an3* mutants (Ercoli et al. 2018). Interestingly, SCARECROW also regulates expression of Arabidopsis telomerase as well as STN1 and CTC1, the core components of the CTC1/STN1/TEN1 (CST) telomere replication complex. Loss of any of these factors causes root developmental abnormalities and stem cell defects (Wang et al. 2023; Surovtseva et al. 2009; Leehy et al. 2013; Song et al. 2008). Furthermore, *an3* mutation decreases cell number and size in the shoot apical meristem (SAM) (Lee et al. 2009; Kinoshita et al. 2020). Finally, telomere length correlates with meristem activity and stem cell development in Arabidopsis (Gonzalez-Garcıa et al. 2015). Overall, these data point to a conserved functional role of genes regulating telomere length and protecting telomere integrity in stem cell maintenance (Hashimura and Ueguchi, 2011; Wang et al. 2023).

Progressive telomere shortening can ultimately lead to genome instability and profound developmental defects. If *A. thaliana* telomeres fall below 1 kb in length, they are recruited to end-to-end chromosome fusions to form dicentric chromosomes which in mitotic cells can be cytogenetically visualized as anaphase bridges (Riha et al. 2001; Heacock et al. 2004). Although telomere length in *oli* and *an3* mutants remained substantially above the length threshold (1.7–2.2 kb), we observed multiple instances of chromatin bridges in late generation oli5 (7%) and oli7 (5.8%) mutants, as well as rare cases of lagging chromosomes in *an3* and *oli2* mutants. However, TF-PCR indicated that telomeres were not engaged in covalent end-joining events. We speculate that these chromosomal segregation defects do not reflect telomere dysfunction, but rather impairment of ribosome biogenesis. Misregulation of ribosome biogenesis processes is known to have an impact on genome stability and aging processes (Kobayashi 2014; Lindström et al. 2018), as well as on cell growth and proliferation (Orsolic et al. 2015, Funk et al. 2016). The incidence of anaphase bridges in *oli5* and *oli7* mutants is similar to sixth generation (G6) telomerase-deficient *tert* mutants, in which up to 6% of all anaphases are defective (Riha et al. 2001). However, while telomeres continue to shorten in the absence of telomerase causing worsening telomere dysfunction in subsequent generations (37% anaphase bridges in G7 tert and up to 50% in G8), telomere length in *oli* mutants stabilizes at a new shorter setpoint, preventing further decline in telomere protection.

OLI2/NOP2, RPL5 and AN3 proteins have conserved key roles in ribosome biogenesis, transcription, and chromatin remodeling throughout eukaryotic evolution (Bourgeois et al. 2015; Mathieu et al. 2003; Vercruyssen et al. 2014). Besides these well-established functions, human RPL5 and SYT (the AN3 homolog) have additional non-canonical roles in tumor suppression (Fancello et al. 2017; Ma et al. 2022; Perani et al. 2003). Similarly, our data uncovered an important and unanticipated role for the *AN3* gene as a positive regulator of *Arabidopsis* telomere length, as well as additional roles of *OLIGOCELLULA* genes in plant chromosome maintenance. The high degree of evolutionary conservation in telomere maintenance mechanisms (Shakirov et al. 2022) suggests these proteins may also contribute to chromosome stability and telomere maintenance in a broad range of eukaryotes.

## Materials and methods

### Plant material, growth conditions, and PCR genotyping.

Seeds for Arabidopsis lines *an3–5* (SALK_208386), *an3–6* (SALK_150407*), stn1–1* (SALK_023504) (Song et al. 2008), *oli2–4/nop2a-4* (SAIL_1279_H03) (Abdulkina et al. 2019), *oli2–2/nop2a-2* (SALK_129648) (Fujikura et al. 2009), *oli5–2/rpl5a-2/ae6–2* (Fujikura et al. 2009), *oli5–3* (SALK_023075), *oli7–2/rpl5b-2* (Yao et al. 2008), and wild type Columbia ecotype (Col-0; CS6673) were obtained from the ABRC stock collection. Seeds were vernalized for three days in 4°C, sterilized in 50% bleach with 0.5% Triton X-100, and plated on MS medium containing 50% Murashige and Skoog Basal Medium with 0.5% agar and 1% sucrose. Seedlings with two true leaves were transplanted into the soil (3:1 ratio of Pro-Mix Bio-fungicide and Profile Field and Fairway Calcined Clay) and grown at 22°C with 16 h light/8 h dark photo period. Genotyping was performed using leaf DNA as previously described (Chastukhina et al. 2016). Primers used for genotyping and for other assays are listed in Supplementary Table 1.

### Telomere length and telomerase activity analyses.

Telomere length analysis was carried out by non-radioactive or radioactive telomere restriction fragment (TRF) methods (Nigmatullina, et al. 2016; Fitzgerald et al, 1999). Genomic DNA from individual whole plants was extracted using the CTAB method (Doyle and Doyle, 1990). DNA was digested with Tru1I (ThermoFisher Scientific) restriction enzyme, resolved in 0.8% agarose gel, transferred to nylon membrane (Hybond N+) and hybridized with 5’ end labeled [T_3_AG_3_]_4_ probe. Telomeric signals were scanned using either the Pharos FX Plus Molecular Imager (Bio-Rad Laboratories) or the Molecular Imager Chemidocs XRS + with Image Lab^™^ Software (Bio-Rad). Data were visualized by Quantity One v.4.6.5 software (Bio-Rad), and mean telomere length (mean TRF) was calculated using the TeloTool program (Gohring et al. 2014).

Relative telomerase activity was measured using quantitative telomeric repeat amplification protocol (qTRAP) as previously described (Song et al, 2019). 3–5 inflorescent bundles from individual plants were ground in liquid nitrogen and resuspend in buffer W+ (1M Tris–Acetate pH 7.5, 1 M MgCl_2_, 2 M Kglutamate, 0.5 M EGTA, 1.5% PVP, 10% glycerol, 1 μM DTT, 0.6 nM RNAse inhibitor (Sintol E-055), 1 μM Protease Inhibitor Cocktail (Sigma)). Protein concentration was measured by DC™ Protein Assay (Bio-Rad). Primer extension step was performed with primer qTRAP-F for 45 min in the dark at room temperature, followed by the qPCR step using SsoAdvanced Universal SYBR Green Supermix (Bio-Rad) with primer qTRAP-R. The average level of telomerase activity in the wild type plants (Col-0) was set as 1. At least three biological replicates (3–5 flower bundles from one plant per replicate) with three technical replicates were performed for each analyzed plant line (Supplemental Data 1).

### Cytogenetic and telomere fusion PCR assays.

For mitotic analysis, spreads were prepared from pistils and stained with DAPI (40,60-diamidino-2-phenylindole), as previously described (Riha et al. 2001). Spreads were imaged at 63X in oil using the fluorescent microscope LSM 780 (Zeiss, Germany). Telomere fusion analysis was performed by PCR using 2 μg of genomic DNA and specific subtelomeric primers as previously described (Heacock et al. 2004). For the extension step, ExTaq polymerase (Takara) was used, and PCR products were resolved in 1% agarose gel with further transfer to the membrane and hybridization with the telomeric probes as described for the TRF analysis. The presence of fused chromosomes was analyzed using subtelomeric primers specific to chromosome arms 1L, 1R, 2R, 3L, 3R, 4R, 5L and 5R, as described before (Heacock et al. 2004).

### Root cell death assay.

After germination, seedlings were grown vertically on MS media plates for 5 days, and intact roots were collected and transferred to Propidium Iodide solution (10 mg/ml in MilliQ water) for 30 sec and rinsed with MilliQ water as described (Bose et al., 2020). Roots were then transferred to slides in a droplet of H_2_0, sealed with a cover slip and imaged at 40X with dsRED filter using a fluorescent microscope LSM 780 (Zeiss, Germany). Only roots with at least a single PI-stained cell in the quiescent center of the root stem cell niche were counted as evidence of cell damage in RAM.

## Statistical analysis

Statistical analysis for telomere length was carried out with GraphPad Prism 8 software using unpaired t-test with Welch correlation. The difference in the values between the wild type (control) and the mutant (experiment) was considered significant for p ≤ 0.05. For the qTRAP experiment, relative telomerase activity was normalized to the average Cq value (meanCq) of a positive control (wild type) using the log2(Cq-meanCq) formula. For analysis of cell death in RAM, statistical significance was calculated using Fisher exact test with the GraphPad Prism 8 software.

## Data Availability

The data that support the findings of this study are available in the supplementary material of this article.
